# Novel pathogenic *VPS13A* mutation in Moroccan family with Choreoacanthocytosis: a case report

**DOI:** 10.1186/s12881-020-0983-8

**Published:** 2020-03-04

**Authors:** Fatima Ouchkat, Wafaa Regragui, Imane Smaili, Hajar Naciri Darai, Naima Bouslam, Mounia Rahmani, Adyl Melhaoui, Yasser Arkha, Elmostafa El Fahime, Ahmed Bouhouche

**Affiliations:** 1 Research Team in Neurology and Neurogenetics, Genomics Center of Human Pathologies, Faculty of Medicine and Pharmacy, University Mohammed V, Rabat, Morocco; 2 Research Team in Neuroncology and Functional Neurosurgery, Faculty of Medicine and Pharmacy, University Mohammed V, Rabat, Morocco; 3 Assistance Units for Scientific and Technical Research (UATRS, CNRST), Allal Fassi/FAR corner, BP 8027, HayRiad, 10000 Rabat, Morocco

**Keywords:** Choreoacanthocytosis, *VPS13A* gene, Nonsense mutation

## Abstract

**Background:**

Choreoacanthocytosis (ChAc), is a rare neurodegenerative disease, characterized by movement disorders and acanthocytosis in the peripheral blood smears, and various neurological, neuropsychiatric and neuromuscular signs. It is caused by mutations in *VPS13A* gene with autosomal recessive pattern of inheritance.

**Case presentation:**

Here we report two patients belonging to a consanguineous Moroccan family who present with movement disorder pathology. They were suspected to have choreoacanthocytosis according to biological, clinical and radiological finding. Thus, whole-exome sequencing was performed for precise diagnosis and identified a homozygous novel nonsense mutation c.337C > T (p.Gln113*) in exon 5 of *VPS13A* in the two affected siblings.

**Conclusion:**

Here, we report a novel nonsense p.Gln113* mutation in *VPS13A* identified by whole-exome sequencing, which caused ChAc in a Moroccan family. This is the first description of ChAc in Morocco with genetic confirmation, that expands the mutation diversity of *VPS13A* and provide clinical, neuroimaging and deep brain stimulation findings.

## Background

Choreoacanthocytosis (ChAc) is a neurodegenerative disease that clinically resembles other pathologies such as Huntington’s disease-like 2, McLeod’s syndrome, pantothenate kinase-associated neurodegeneration, all grouped into the Neuroacanthocytosis syndrome [1, 2]. ChAc is characterized by onset at adult age, often between 30 and 40 years and by the combination of movement disorders due to degeneration of basal ganglia and acanthocytosis in the peripheral blood non often reported as a beginning symptom but may appear late during the disease course. Clinically, ChAc is heterogeneous and presents mainly with chorea, parkinsonism, dystonia, tics and involuntary movements. Self-mutilation such as tongue and lip biting are common and can be considered pathognomonic of the disease [3, 4]. Other symptoms as epilepsy, neuropsychiatric and neuromuscular symptoms may be added [5], and sometimes it can be in an atypical form presenting as epilepsy [6] or obsessive-compulsive behaviour [7].

ChAc was mapped to chromosome 9q21 in a 6 cM interval [3], in which the causative gene was identified, the vacuolar protein sorting 13A gene (*VPS13A*) comprising 73 exons and encoding a 360-kDa protein named chorein [8]. ChAc follows an autosomal recessive inheritance pattern and fifty-five mutations were reported up to date as pathogenic or likely pathogenic in the *VPS13A* gene on the Clinvar web site (ncbi.nlm.nih.gov/clinvar) from which 29 were duplications, 17 INDEL mutations and 9 were single nucleotide variations (SNV). These mutations have been reported in approximately 1000 patients worldwide [9], but never in the African continent.

Here, we report a Moroccan family with two patients displaying clinical heterogeneity of ChAc and in whom we identified a novel nonsense mutation (p.Gln113*) in *VPS13A* by whole exome sequencing (WES).

## Case presentation

### Clinical information

All the studies were carried out after approval of the local ethical committee of biomedical research (CERB). A written informed consent was obtained from all of the participants in the study.

**Fig. 1:**
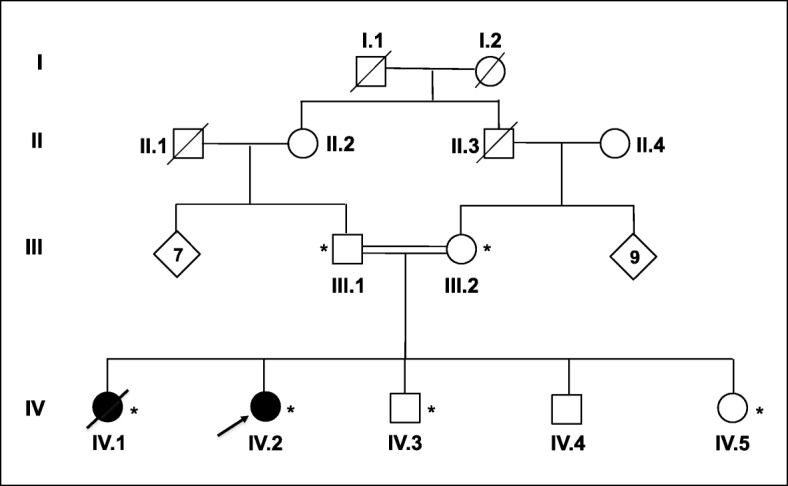
Pedigree of a consanguineous Moroccan family with ChAc. Arrow: index patient. Asterisk: genetic testing performed

A consanguineous family of Moroccan Origin, with two affected siblings with suspicion of a movement disorder pathology, was recruited in the Department of Neurology and Neurogenetics, Specialities Hospital of Rabat (Morocco). The pedigree of the family is shown in Fig. 1.

**Fig. 2:**
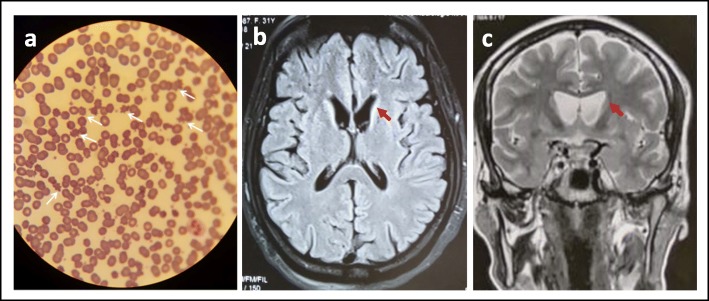
Biological and neuroimaging features of patient IV.2 presenting with choreoacanthocytosis. **a** Acanthocytes on peripheral blood smear (white arrows); **b, c** Atrophy of the caudate nucleus head on axial and coronal view respectively (red arrows)

The index patient IV.2 is a 32-year-old woman who presented during the last 2 years gradual onset of involuntary upper limb choreiform movements spreading to the whole body with trunk spasms and gait disturbance. Symptoms have worsened few months later by the appearance of oromandibular dystonia as jaw-opening dystonia, tongue protrusion hindering her from eating and talking. Patient developed later self-mutilation by biting of the tongue, lips and forearms, vocalizations and psychiatric disorders such as depression and compulsive behaviours. Neurological examination showed otherwise areflexia, generalized hypotonia and scars of lip and forearm biting. The score of mini-mental status examination was 22/30 and Montreal cognitive assessment was 19/30 showing cognitive impairment especially frontal lobe dysfunction. Sixty percent of acanthocytes were detected in peripheral blood smear test (Fig. 2a), and serum CK, LDH, AST and ALT levels were normal. Magnetic resonance imaging (MRI) showed bilateral and symmetrical striatal atrophy (Fig. 2b-c) and electroneuromyography (ENMG) was normal. The proband received Haloperidol 1,5 mg three times daily, trihexyphenidyl 5 mg three times daily and baclofen 10 mg four times daily for 1 year but without improvement. Therefore, a bilateral GPI DBS has been performed because of the dramatic worsening of the symptoms and the ineffectiveness of drugs. A permanent quadripolar leads were implanted (model 3389; Medtronic, Minneapolis, Minn., USA), with the following stimulation parameters: right case (+), contact 0 (−); amplitude 2 V; pulse-width 60 μs; frequency 130 Hz; left case (+), contact 8 (−); amplitude 2 V; pulse-width 60 μs; frequency 130 Hz. At the 2 months follow up review, a response rate of 60% was expressed by the patient and her family. The tongue protrusion disappeared allowing the patient to eat on a sitting position, and to speak more clearly. The self-injuring has lessened to only the lip biting. We also notice a gain in posture, gait and in the sitting ability. However, the foot dystonia persisted. She is more autonomous, able to perform domestic chores alone.

The older sister IV.1, 41-year-old, suffered since the age of 36, from generalized epileptic seizures stabilized with with a daily 1500 mg of valproic acid. She presented since 3 years gait disturbance and psychiatric features, depression and apathy. Neurological examination revealed a mild resting tremor of the right arm, akinesia, generalized hypotonia, areflexia and bilateral foot dystonia. The electroencephalogram was normal but MRI and ENMG were not possible because the patient subsequently died from soft-tissue tumor complications.

### Whole exome sequencing and data analysis

Blood samples were taken from patients IV.1 and IV.2 and family members III.1, III.2, IV3 and IV.5, and high quality genomic DNA was purified from peripheral blood leukocytes using Wizard® Genomic DNA Purification Kit from Promega.

Quality and concentrations of DNA samples were examined by Qubit dsDNA HS Assay Kit (Invitrogen, Q32851) on Qubit fluorometer (Invitrogen, Q33216). WES was performed in the patient IV.2 and his father III.2 at the Supporting Units for Technical and Scientific Research Belonging to CNRST (UATRS/CNRST, Rabat, Morocco). The target regions in the exome were amplified using Ion Proton platform (Thermo Fisher, Scientific). Library preparation, bead templating and PI Chips v2 loading were performed on Ion Chef System, and the sequencing was done on the Ion Proton machine. Sequence alignment to Hg19 and variant identification were performed with the Torrent Suite v.4.2.1 software. The generated VCF files were then imported into the online Server of Ion Reporter Software v5.6 for variant analysis and annotations. Sanger sequencing was used to validate the significant variant identified by WES using a SeqStudio automated sequencer (Thermo Fisher Scientific). Co-segregation analyses among patients’ family were also performed.

**Fig. 3:**
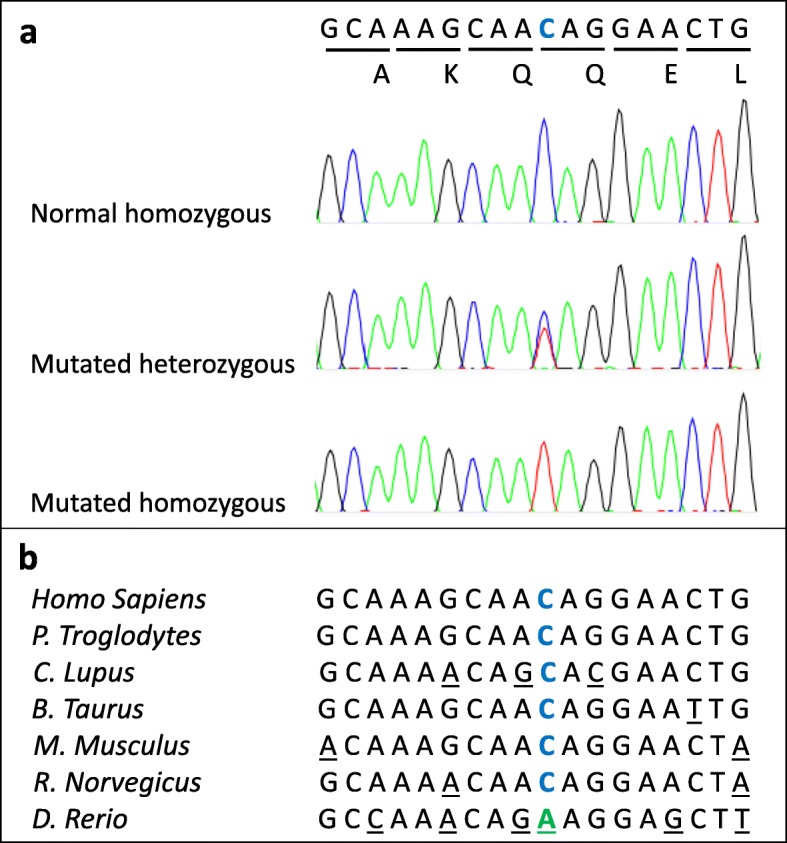
Sanger validation and conservation of the novel discovered *VPS13A* nonsense mutation. **a** Sanger sequencing confirms the presence of the Gln113* mutation in *VPS13A* at heterozygous and homozygous state; **b** Partial amino acid sequence alignment of human *VPS13A* with orthologs shows evolutionary conservation between species of the Glutamine residue

Analysis of WES data showed that 94 % exons were covered by at least 40X. The primary filtering by Ion Reporter software led to the identification of 39,880 variants in 13,566 genes consisting of SNVs, MNVs and INDELs. These mutations were subsequently filtered using the zygosity in homozygous filter that yielded 3540 homozygous variants in 2836 genes. A novel homozygous c.337C > T mutation was found in exon 5 of *VPS13A* gene leading to a p.Gln113* nonsense mutation. The WES data showed that the mother III.2 was heterozygous for this variant. The p.Gln113* variant was absent from the gnomAD database of control individuals. This nonsense mutation was validated by Sanger sequencing in all the family members (Fig. 3a) showing a co-segregation of the mutation with the disease (the two patients were homozygous and the parents and the brother IV.3 were heterozygous, the sister IV.5 was homozygous for the normal allele), absent from 192 ethnically matched controls, affect a residue highly conserved among species (Fig. 3b) and was therefore considered as pathogenic.

## Discussion and conclusion

In this study, we present two ChAc patients belonging to a Moroccan consanguineous family by describing the clinical symptoms, neuroimaging characteristics and genetic findings.

The clinical phenotype of the two studied patients strongly evoked ChAc disease due to mutations in *VPS13A* gene. Since this gene is a large and contains 73 exons, it is difficult to explore by traditional Sanger sequencing. WES has become a powerful tool for identification of disease-causing variants in genetic disease when the clinical phenotype does not evoke a known disease or when the suspected causal gene is large, thus allowing to save a lot of time and money. WES using the Ion Proton technology found a novel homozygous nonsense mutation c.337C > T (p.Gln113∗) in exon 5 of *VPS13A* in the proband. The mutation we validated by Sanger sequencing and segregated in all the family members according to a recessive mode of inheritance confirming the diagnosis of the ChAC in the two patients.

*VPS13A* encodes the protein chorein is expressed ubiquitously in the brain and in many other tissues [10]. It is composed of multiple domains, a long N-terminal region (VPS13α), a putative WD40 modules, a domain reminiscent of a DH domain and a C-terminal pleckstrin homology (PH) domain [11]. The underlying pathophysiology of ChAc is not yet well known, but recently it was shown that chorein may have a role in lipid exchange between organelles and thus for membrane lipid homeostasis in the nervous system. In deed, it has shown that N-terminal region of Vps13A, called Chorein_N domain, is a lipid transport module that bind to the endoplasmic reticulum and connects it to mitochondria [12]. The p.Gln113* identified in this study is located in the chorein_N domain causing loss of function due to truncated protein.

Clinically, the index patients presented hyperkinetic movements with self-injuring which are the main symptoms in choreoacanthocytosis and can be considered as pathognomonic to the diagnosis [4]. On the other hand, the proband’s sister presented generalized epilepsy as initial symptom and later parkinsonism which was previously described in patients with a long follow up when the degenerative process reaches the nigrostriatal pathways [4, 13]. This phenotypic variability has been already documented in many cases of ChAc even in twins [5] and suggests the implication of other modifier genes, epigenetic and environmental factors on the pathogenesis of neuroacanthocytosis as discussed elsewhere [14].

The clinical phenotype of both patients is enriched by depression and emotional liability. Indeed, psychiatric features account for 60% of ChAc cases including depression, schizophrenia, paranoia, obsessive–compulsive disorder and emotional liability [15]. Beside the psychiatric symptoms, the neuropsychological tests revealed impaired executive and visuospatial functions in the index patient. Ichiba M et al. [16] described a radiological correlation to the cognitive deficit due to the mild atrophy of the frontal lobe and the decrease of brain perfusion on SPECT brain imaging, in addition to the atrophy of the head of caudate nucleus.

Nowadays, there is no curative treatment that can stop the progression of the disease, it remains symptomatic using drugs such as new generation antipsychotics and antiepileptic drugs. Botulinum toxin is indicated in perioral dystonia with a proven effect [17] but there are no evident recommendations concerning protocol, site of injection and doses.

There are a few reported cases describing the effectiveness of deep brain stimulation (DBS) in managing movement disorders in choreoacanthocytosis. The globus pallidus pars interna (GPi) remains the most used target requiring low frequency stimulation on patient with prominent chorea and high frequency stimulation on notable dystonia. The combination of GPi and the ventralis oralis complex of the thalamus gave better results especially improvement of hyperkinetic movements with less side effects [18]. In the studied family, a GPi DBS was done recently in the proband because of the dramatic worsening of her symptoms and the ineffectiveness of drugs. After 2 months follow up, the patient showed the improvement of hyperkinetic movement and trunk spasm and a mild amelioration of dysarthria, her speech being more intelligible, whereas foot dystonia persisted. Further follow up are necessary to assess the real effect of DBS on this last symptom. These results are in accordance with a multicenter retrospective study that scrutinized the short and long term outcome of DBS in 15 patients [19]. Indeed, chorea, orofacial dyskinesia and trunk spasm were the first improved symptoms, immediately after switching on stimulation. The effect was delayed in dystonia and disappointing for dysarthria which is less sensitive to GPi-DBS or even worsened by high frequency stimulation [20].

In summary, we highlight the ChAc clinical heterogeneity in two patients belonging to a Moroccan consanguineous family, in which we identified a novel nonsense mutation c.337C > T in *VPS13A* gene. This mutation results in the production of a truncated protein with 113 amino acids (p.Gln113*).

## Data Availability

The datasets used and analyzed during the current study are available from the corresponding author upon request. The novel *VPS13A* mutation was submitted to ClinVar under accession number: SCV001147072.1.

## Abbreviations

CERB: Ethical committee of biomedical research
ChAc: Choreoacanthocytosis
CNRST: Centre national de recherche scientifique et technique
DBS: Deep brain stimulation
ENMG: Electroneuromyography
GPi: Globus pallidus interna
MRI: Magnetic resonance imaging
SNV: Single nucleotide variations
VPS13A: Vacuolar protein sorting 13 homolog A
WES: Whole exome sequencing
